# Supplementary figures and images for: Intranasal parainfluenza virus-vectored vaccine expressing SARS-CoV-2 spike protein of Delta or Omicron B.1.1.529 induces mucosal and systemic immunity and protects hamsters against homologous and heterologous challenge
Source: PLoS Pathog. 2025 Apr 21;21(4):e1012585. doi: 10.1371/journal.ppat.1012585 (PMC12054915; doi:10.1371/journal.ppat.1012585)

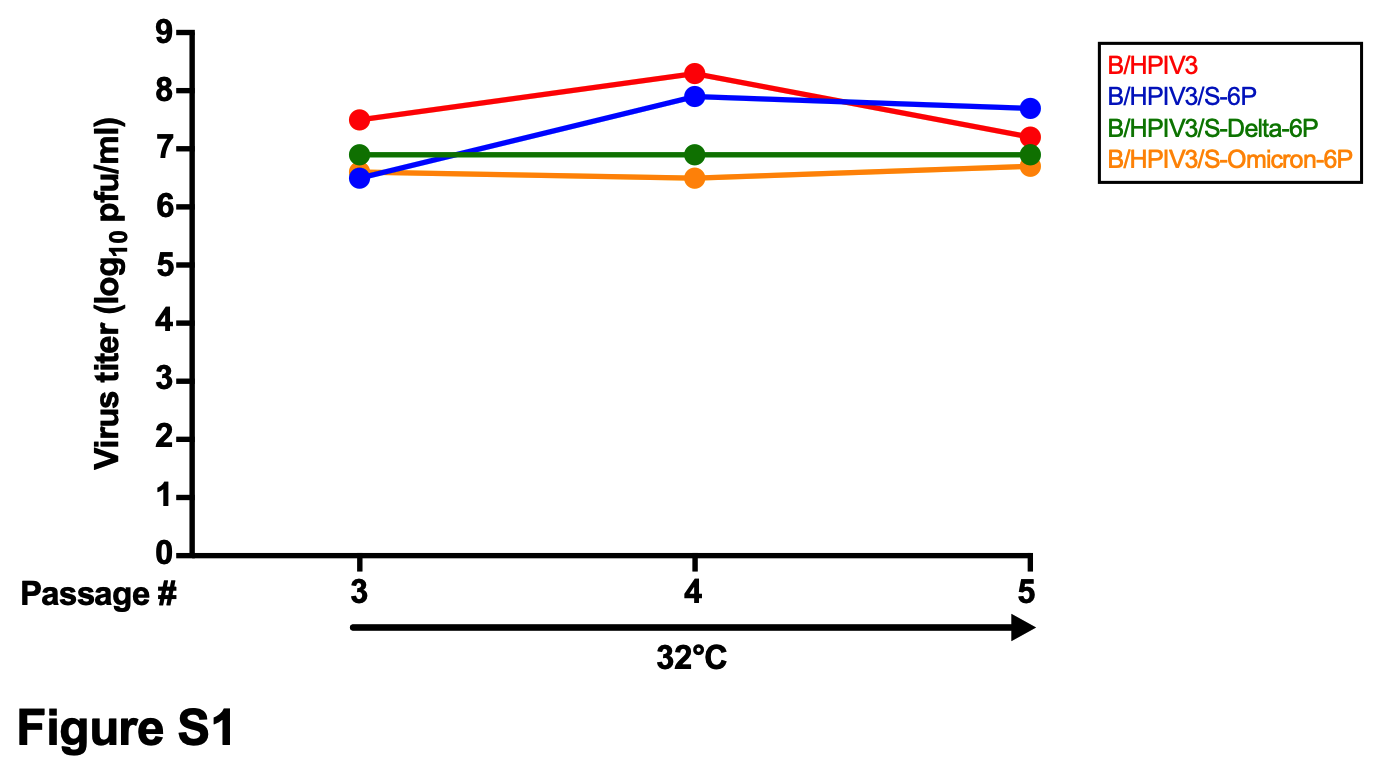

Supplement: S1 Fig — Monolayers of Vero cells in 25 cm2 flasks were inoculated with sequenced working stocks (corresponding to passage 2) of B/HPIV3, B/HPIV3/S-6P, B/HPIV3/S-Delta-6P or B/HPIV3/S-Omicron-6P using an initial MOI of 0.01 PFU/cell and incubated at 32°C. On day 6 post-infection, corresponding to the peak of virus replication [25], supernatant from each flask was harvested, aliquoted and snap-frozen in dry ice and stored at -80°C. Then, 1 ml of each virus supernatant was used to infect a new monolayer of Vero cells in 25 cm2 flasks and this procedure was repeated one more time for a total of three serial passages of the initial working stocks. At the end of the experiment, virus titers after each passage were determined by an immunoplaque assay. (TIF) [file ppat.1012585.s002.tif]

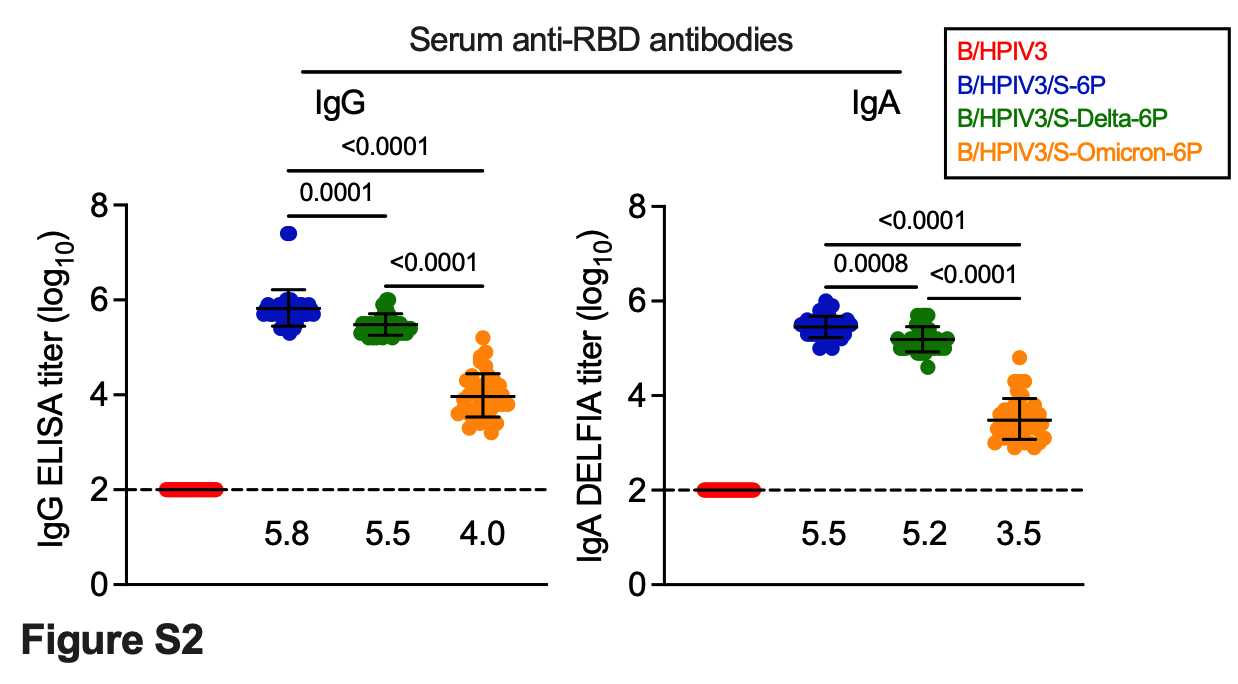

Supplement: S2 Fig — On day 24 or 25 pi, serum was collected from n = 36 hamsters per group. Anti-RBD IgG (left panel) and IgA (right panel) serum antibody titers were evaluated by ELISA using purified RBD antigen preparations specific for the Wuhan-Hu-1 strain. IgG and IgA titers using purified S antigens matching the WA1/2020 or B.1.617.2/Delta or B.1.1.529/Omicron variants are shown in Fig 2B. Each hamster is represented by a symbol, and medians with interquartile ranges are shown. The limit of detection (dotted line) is 2 log10. One-way ANOVA with Sidak post-test; exact p values are indicated for levels of significance p < 0.05. (TIFF) [file ppat.1012585.s003.tiff]

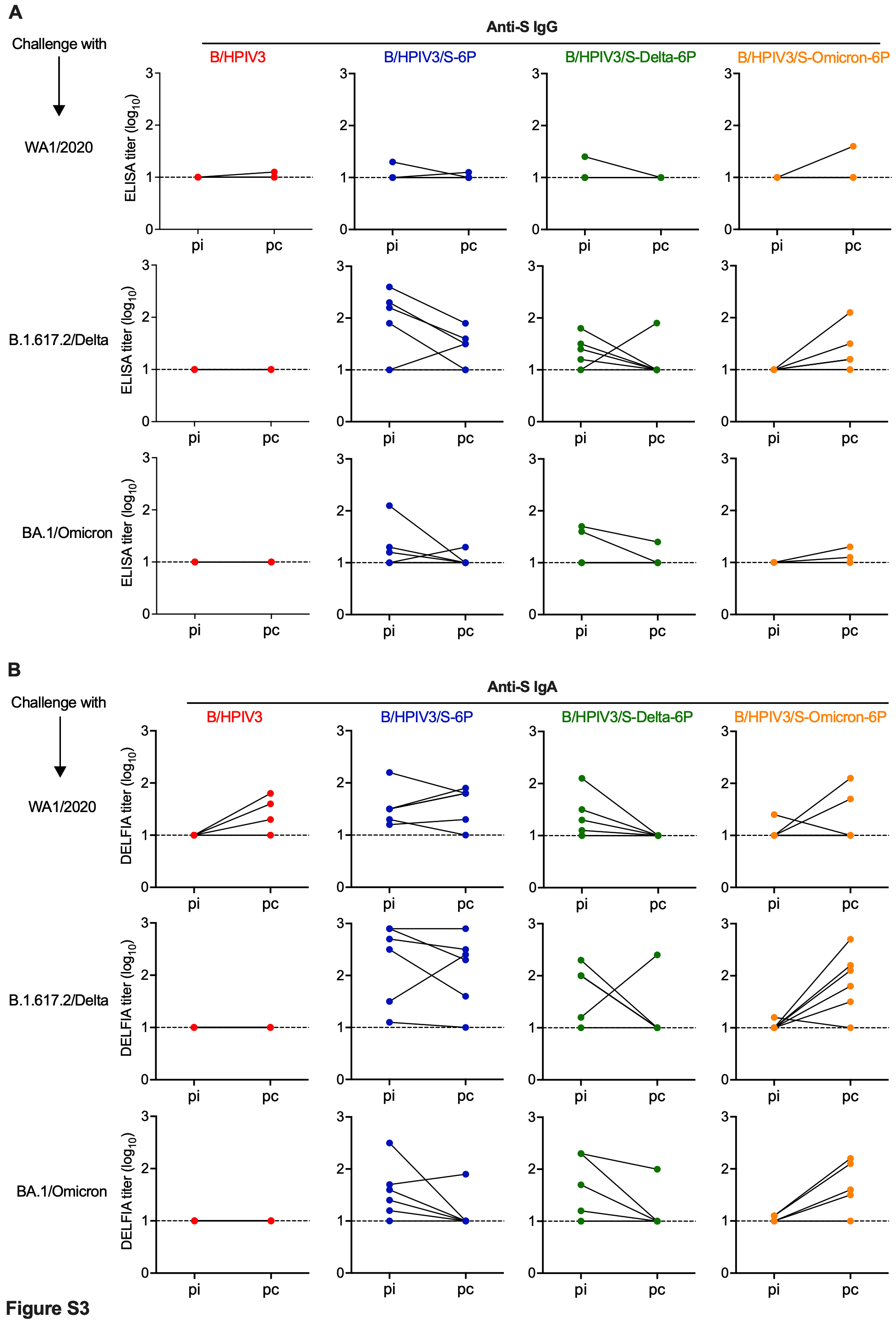

Supplement: S3 Fig — On day 21 post-immunization (pi), nasal washes were performed on 18 hamsters per immunized group, picked at random. On day 21/22 post-challenge (pc; equivalent to day 53/54 pi), nasal washes were performed on the six remaining hamsters per subgroup (see Fig 1C for timeline of experiment). Anti-S IgG (A) and IgA (B) titers of paired nasal wash samples from the same six hamsters per subgroup were determined by ELISA. Note that ELISA titers from the samples collected on day 21 pi are also included in the results from 18 animals shown in Fig 2A. Each hamster is represented by a symbol. The limit of detection of ELISA titers is 1 log10. (TIF) [file ppat.1012585.s004.tif]

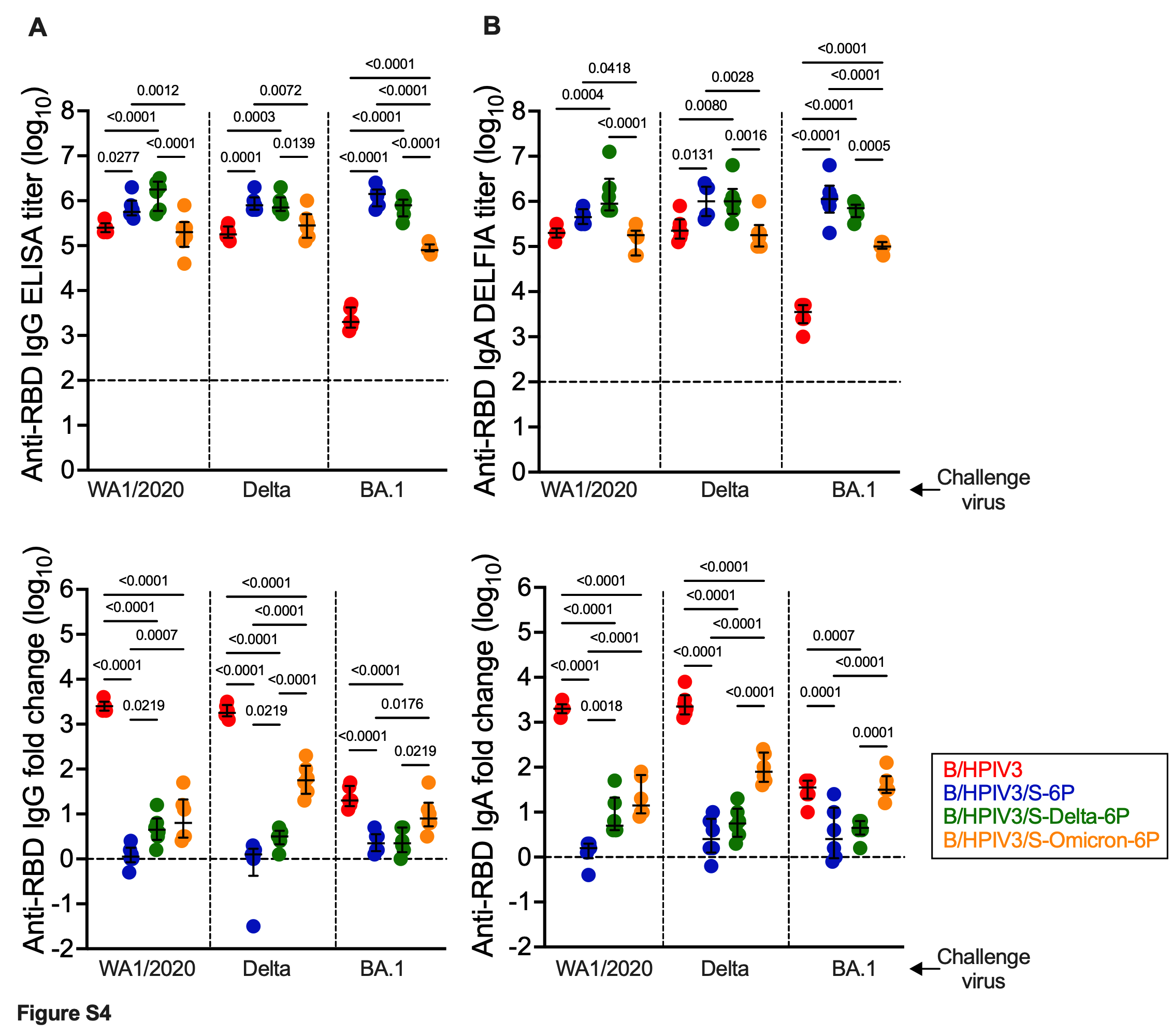

Supplement: S4 Fig — On day 23 or 24 pc, the six remaining hamsters per subgroup were euthanized, and sera were collected (see Fig 1C for timeline of the experiment) for evaluation of the antibody response by ELISA. Serum anti-RBD IgG (A) and IgA (B) titers after challenge (top panels) and fold changes of post-challenge titers over post-immunization titers (bottom panels). N = 6 with the exception of n = 5 for B/HPIV3-immunized/WA1/2020 challenged. Purified preparations of RBD from the Wuhan-Hu-1 strain were used as an antigen in the ELISA. Each hamster is represented by a symbol and medians with interquartile ranges are shown. One-way ANOVA with Tukey post-test; exact p values are indicated for levels of significance p < 0.05. (TIFF) [file ppat.1012585.s005.tiff]
